# The effect of systemic acetazolamide administration on intraocular pressure in healthy horses—A preliminary study

**DOI:** 10.1111/vop.13240

**Published:** 2024-06-05

**Authors:** Anat Shnaiderman‐Torban, Oren Pe'er, Kajsa Gustafsson, Amos Tatz, Malka Brizi, Stefan Soback, Wiessam Abu Ahmad, Ramon Magen, Ron Ofri, Gal Kelmer

**Affiliations:** ^1^ Koret School of Veterinary Medicine (KSVM), The Robert H. Smith Faculty of Agriculture, Food and Environment The Hebrew University of Jerusalem Rehovot Israel; ^2^ Department of Veterinay Medicine and Animal Sciences University of Milan Lodi Italy; ^3^ Kimron Veterinary Institute Bet Dagan Israel; ^4^ Hadassah Braun School of Public Health and Community Medicine The Hebrew University of Jerusalem Jerusalem Israel

**Keywords:** carbonic anhydrase inhibitors, equine glaucoma

## Abstract

**Objectives:**

In equine glaucoma, topical treatment with carbonic anhydrase inhibitors (CAIs) is recommended. Oral acetazolamide, a systemic CAI, is used in horses with hyperkalemic periodic paralysis. Information regarding its effect on equine intraocular pressure (IOP) is scarce. The aim of the study was to determine the effect of oral acetazolamide treatment on IOP in horses, in a case–control study.

**Animals:**

Ten healthy horses.

**Procedures:**

Horses were treated with oral acetazolamide (4.4 mg/kg) BID for 1 week. Serum acetazolamide concentrations were determined by liquid chromatography/tandem mass spectrometry, and IOP were measured before treatment, daily during treatment, and at 48 and 72 h after treatment.

**Results:**

Acetazolamide serum levels reached steady state at 72 h after the first oral dose. In a mixed effect model logistic regression, there was a significant decrease in IOP on the third treatment day, of 2.4 mmHg (*p* = .012) and 2.7 mmHg (*p* = .006) in the left (OS) and right eye (OD), respectively. On the seventh day, there was a decrease in 2.5 mmHg (*p* = .008) and 2.7 mmHg (*p* = .007) OS and OD, respectively. A significant increase occurred 48 h following treatment discontinuation (3.6 mmHg, *p* < .001 and 3.5 mmHg, *p* < .001 OS and OD, respectively). The area under the concentration versus time curve (AUC_(0–10h)_) was 1.1 ± 0.5 μg/mL*h, mean residence time 6.7 ± 4.3 h, peak plasma concentration (*C*_max_) 0.4 ± 0.4 μg/mL and time to reach *C*_max_ 1.8 h. There was a significant increase in serum concentrations 1, 2, 48, 72, and 156 h following the first drug administration (*p* < .05).

**Conclusions:**

Further studies are required to determine whether acetazolamide is a potential treatment for equine glaucoma.

## 1 INTRODUCTION

Glaucoma is a multifactorial and neurodegenerative disease that damages the retina, optic nerve, and has a poor prognosis for vision.^1^ In horses, glaucoma is usually secondary to chronic uveitis, especially equine recurrent uveitis.^1^, ^2^, ^3^ The main treatment for glaucoma in horses includes drugs designed to lower intraocular pressure (IOP) by targeting the production of aqueous humor, the pressure in the episcleral veins, and/or drainage of aqueous humor through the trabecular or uveoscleral outflow pathways, in addition to anti‐inflammatory treatment. A combination of topical carbonic anhydrase inhibitors (CAI) and β‐blockers is the most effectively used formulation in horses. Surgical treatment, including cyclocryosurgery, cyclophotocoagulation, or gonioimplants, is indicated in cases that are nonresponsive to pharmaceutical therapy.^1^

In humans, dogs, and cats, a systemic CAI, acetazolamide, is considered an effective drug for glaucoma.^4^ It is an oral diuretic, which reduces IOP by reducing the aqueous humor production in the ciliary body, and is considered effective in IOP lowering. However, clinical use is limited due to its side effects, including neurological, renal, hematological, metabolic, and gastrointestinal disorders, which were reported in humans^4^ and companion animals.^4^ Nonetheless, acetazolamide is used to treat hyperkalemic periodic paralysis (HYPP) in horses, with an established dosage of 2.2–4.4 mg/kg twice daily.^5^ Single‐dose pharmacokinetics of acetazolamide administered intravenous and orally has been studied in horses^6^ as well as its effect on gas exchange and acid–base control in pulmonary circulation in exercising horses.^7^, ^8^ However, its use in systemic treatment of glaucoma has not been investigated, and the effect of the drug on equine IOP is unknown. A hypotensive effect of acetazolamide may allow this drug to be used in combination with others to better treat equine glaucoma.

The main aim of this study was to determine the effect of acetazolamide on IOP in healthy horses treated with a dose of 4.4 mg/kg twice daily for 1 week. The secondary aims included measurement of serum drug concentrations in acetazolamide‐treated horses as well as the recording of potential systemic side effects.

## 2 MATERIALS AND METHODS

A prospective trial was designed, and the study protocol was approved by the Institutional Animal Care and Use Committee of the Kimron Veterinary Institute (approval number 020_b13108_3).

### 2.1 Animals

Horses were recruited from a horse trader herd. Inclusion criteria included physically and, specifically, ophthalmologically healthy horses that were easy to handle. All horses were local breed adult horses, including two geldings and eight not‐pregnant mares. The estimated ages of participating horses ranged from 2 to 18 years (mean 10 years), and their body weight ranged from 285 to 560 kg (mean 400 kg). Horses underwent a physical exam by an equine internal medicine resident. Complete ophthalmic examination was performed by an ophthalmology resident including menace response, dazzle and pupillary light reflexes, slit‐lamp biomicroscopy (Kowa SL‐15, Portable Slit‐Lamp Biomicroscope), and indirect ophthalmoscopy (Welch Allyn) with a 20 diopter lens (Volk Optical, Inc). All horses were found free from any ocular abnormalities. Complete blood count, urea, creatinine, and electrolyte levels were obtained.

### 2.2 Study design

**FIGURE 1:**
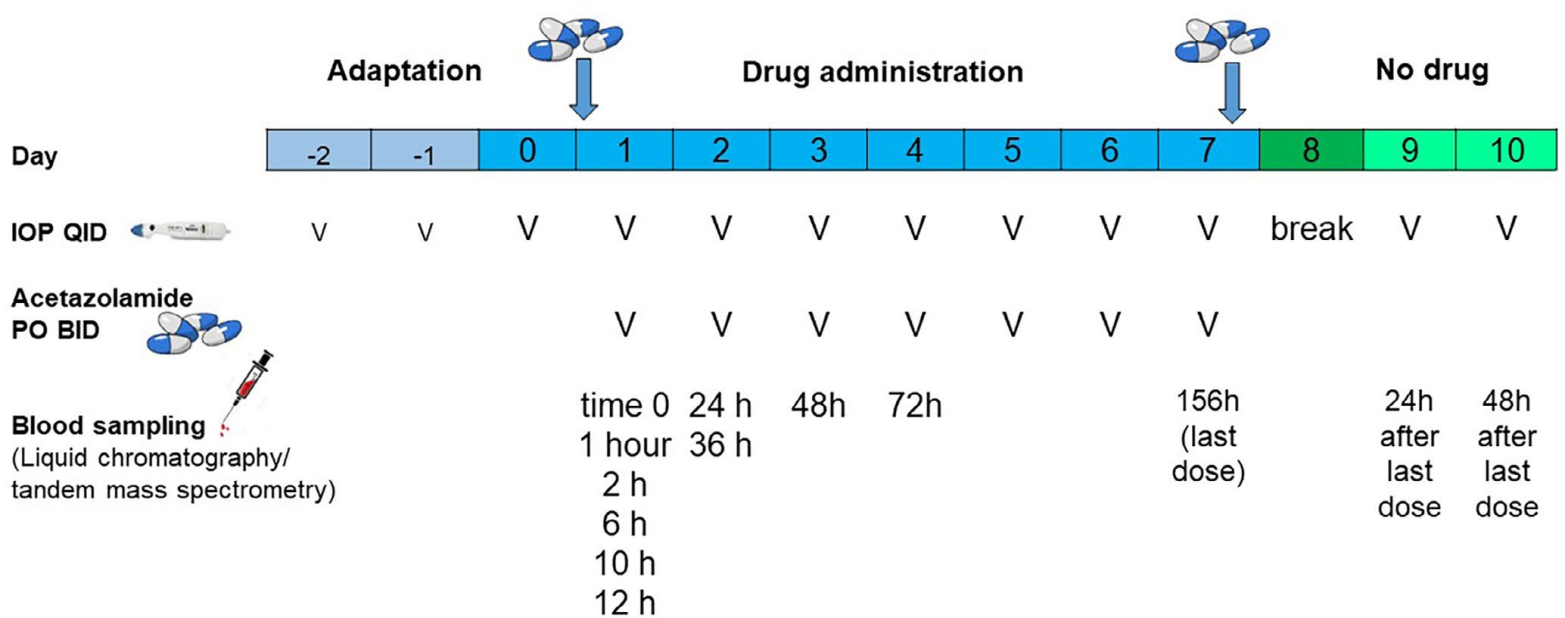
Study design: the first 2 days of the experiment (days minus two and minus one) were acclimation days, during which IOP was measured four times daily (6 a.m., 10 a.m., 14 p.m., and 18 p.m.) in both eyes of 10 horses. On days 1–7, horses were treated with oral acetazolamide at 12‐h intervals. Blood samples were collected seven times on day 1 at time 0 (before first drug administration) and after 1, 2, 6, 10, 12, and 24 h. The concentrations after 12 h, and all subsequent time points were determined in blood samples drawn immediately before administration of the next oral drug dose. Subsequently, blood samples were collected at 36, 48, 72, and 156 (day 7) hours following the first drug administration. On day 8, the horses received no medication, nor were any diagnostic tests performed. On days 9 and 10, IOP was measured four times daily as previously described, and blood samples were collected at 204 and 228 h following the first dose of acetazolamide.

The study design is presented in Figure 1. The first 2 days of the experiment (days minus two and minus one) were acclimation days, during which IOP was measured four times daily (6 a.m., 10 a.m., 14 p.m., and 18 p.m.) in both eyes. Intravenous catheters were inserted during the acclimation period, to sedate horses and collect blood (a polyurethane over the needle catheter, 14 ga × 13 cm, MILA International, Inc. Florence, Kentucky, USA).

For tonometry, horses were sedated with a minimal intravenous dose of xylazine needed for each horse (an average total dose of 20 mg, Thiazine 100 mg/mL, Ceva Animal Health Pty Ltd, Australia). No auriculopalpebral nerve block was administered, the head was kept parallel to the floor, and pressure on the jugular veins was avoided. Tonometry was conducted using a TonoPen (Reichert Technologies, Depew, NY), following instrument calibration and application of topical anesthesia (0.4% oxyburpocaine hydrochloride, Fischer Pharmaceutical Labs., Israel), with the first eye tested determined randomly. Only IOP values measured on day 0 were used to calculate the baseline for statistical analysis. On days 1–7, horses were treated with crushed acetazolamide tablets (Uramox, Taro Pharmaceutical Industries Ltd, Israel), 4.4 mg/kg, at 12 h intervals (at 7 a.m. and p.m.), given orally through a syringe, mixed up with a sweetener. IOP was measured bilaterally as previously described and an average daily IOP was calculated to exclude the circadian IOP variation.

To measure serum drug levels, blood samples were collected seven times on day 1 at time 0 (before first drug administration) and after 1, 2, 6, 10, 12, and 24 h. The concentrations after 12 h, and all subsequent time points, were determined in blood samples drawn immediately before the adminstration of the next oral drug dose. Subsequently, blood samples were collected at 36, 48, 72, and 156 (day 7) hours following the first drug administration. Blood sampling was done immediately before the next drug administration. On day 8, the horses received no medication, nor were any diagnostic tests performed. On days 9 and 10, IOP was measured four times daily as previously described, and blood samples were collected at 204 and 228 h following the first dose of acetazolamide. The blood was allowed to clot for 1 h, and serum was separated by centrifuge and frozen at −80°C pending drug analysis. A physical examination was performed daily by an equine clinician. At the end of the study period electrolytes, creatinine, and urea levels were measured.

### 2.3 Serum sample analysis

Acetazolamide concentration in serum was determined by liquid chromatography‐tandem mass spectrometry (LC–MS/MS). A volume of 50 μL of serum was mixed with 50 μL of bi‐distilled water. Acetonitrile (100 μL) containing 10 ng/mL of chloramphenicol‐d_5_ (internal standard) was added. Samples were vortexed and centrifuged at 16 000 *g* for 5 min at 4°C. Finally, an aliquot of the supernatant was submitted to LC–MS/MS analysis. Acetazolamide was spiked in blank horse serum to produce a calibration curve with a concentration range between 0.025 and 2.5 μg/mL. Intraday and interday accuracy and precision of the analytical method were determined for a low level (LL) of 0.025 μg/mL, medium level (ML) of 0.25 μg/mL, and high level (HL) of 2.5 μg/mL.

The mass spectrometer (API 4000, Applied Biosystems, Toronto, Canada) was connected to a liquid chromatograph (Agilent Technologies Series 1200 system, Waldbronn, Germany), including the modules of a binary pump, degasser, auto sampler, and a thermostatic column compartment.

Chromatographic separation was performed using a Zorbax Eclipse Plus C18 column (1.8 μ, 50 × 4.6 mm; Agilent Technologies, Waldbronn, Germany). A gradient program was operated with formic acid solution, 0.2% (solvent A), and acetonitrile (solvent B) at a flow rate of 1.2 mL/min. The percentage of solvent B was increased from 5% to 70% within 1 min, returned to 5% between 1.5 and 1.7 min and allowed to stabilize to the initial conditions up to 5 min. The injection volume was 3 μL, and the column compartment was maintained at 40°C.

MS/MS detection of acetazolamide was performed by Turbo spray equipped with electrospray ionization (ESI) in negative mode. Multiple reaction monitoring was applied for the measurement of the precursors 220.8 and 325.9 m/z for acetazolamide and chloramphenicol‐d5, respectively. The product ions were 83.1 and 156.9 m/z for acetazolamide and chloramphenicol‐d_5_, respectively.

### 2.4 Pharmacokinetic analysis

After the first administration (0–10 h) analysis was performed using the noncompartmental method using a computer program (PK Solutions 2.0, Summit Research Services, Montrose, CO, USA). The AUC_0–10h_, *C*_min_, mean residence time (MRT), and *t*_1/2_ were determined. Consequently, the *t*_min_ was obtained by visual examination of the data. The observed *t*_1/2_ was used to determine the time to reach a steady state.

### 2.5 Statistical methods

The minimal sample size required for the study was calculated using a paired *t*‐test exam (WinPEPI PairsEtc. Sample size S5). The normal IOP of a horse is 17–28 mmHg.^9^ The standard deviation of the TonoPen is 5 mmHg, therefore, in order to detect a decrease of 5 mmHg, and assuming a standard deviation of the differences of 5 mmHg, 10 pairs of observations would be needed to detect a significant difference at an alpha of .05 and a power of 90%. A pair means one horse (2 eyes), and therefore, 10 horses were included in the study.

Pearson correlation test was performed to evaluate the correlation between both eyes. A mixed effect logistic regression model was selected to determine significant changes in drug serum concentrations and IOP values (Stata Statistical Software: Release 15. College Station, TX: StataCorp LLC).

## 3 RESULTS

### 3.1 Intraocular pressure response

All changes mentioned in IOP are in comparison to the baseline values calculated for each individual horse on day 0. A strong correlation was detected between both eyes (*r* = .8, *p* < .001). A significant decrease in IOP was detected on days 3 and 7 of acetazolamide administration (Table 1). On day 3, a mean decrease of 2.4 mmHg was detected in the left eye (95% CI −4.2, −0.53, *p* = .012), and a mean decrease of 2.7 was detected in the right eye (95% CI −4.6, −0.78, *p* = .006). On day 7, a decrease of 2.5 mmHg was detected in the left eye (95% CI −4.4, −0.7, *p* = .008), and a decrease of 2.7 was detected in the right eye (95% CI −4.59, −0.72, *p* = .007). On day 9 of the experiment, 48 h following treatment discontinuation, a significant increase occurred in both eyes (3.6 mmHg; 95% CI 1.7, 5.4, *p* < .001 in the left eye and 3.5 mmHg; 95% CI 1.6, 5.4, *p* < .001 in the right eye).

**TABLE 1:** The average daily intraocular pressure in healthy horses before, during, and after acetazolamide treatment.

Day^a^	Average OS IOP	OS IOP difference versus day 0 (95% CI)	*p*‐value	Average OD IOP	OD IOP difference versus day 0 (95% CI)	*p*‐value
0	20.23			20.28		
1	19.38	−1.00 (−2.86, 0.86)	.29	18.89	−1.38 (−3.32, 0.56)	.16
2	19.32	−0.91 (−2.77, 0.95)	.34	19.22	−1.05 (−2.99, 0.89)	.29
3	17.85	−2.39 (−4.24, −0.53)	**.012**	17.59	−2.72 (−4.66, −0.78)	**.006**
4	19.29	−0.94 (−2.8, 0.91)	.32	20.01	−0.30 (−2.25, 1.63)	.76
5	19.11	−1.00 (−2.89, 0.82)	.28	20.38	−0.66 (−1.01, 1.87)	.98
6	19.91	−0.37 (−2.22, 1.49)	.7	20.33	−0.26 (−2.2, 1.68)	.79
7	17.73	−2.50 (−4.36, −0.65)	**.008**	17.66	−2.66 (−4.59, −0.72)	**.007**
9	23.78	3.55 (1.69, 5.4)	**<.001**	23.81	3.49 (1.55, 5.43)	**<.001**
10	19.64	−0.59 (−2.45, 1.28)	.54	20.25	−0.06 (−2.01, 1.89)	.95

*Note*: Bolded values were significant.

Abbreviations: IOP, intraocular pressure; OD, “oculus dexter,” right eye; OS, “oculus sinister,” left eye.

^a^ Day 0 is the last day of acclimation period, and readings on that day constituted the baseline IOP values. Day 1 was the first day of drug administration. On days 8–10, horses were not treated with acetazolamide.

### 3.2 Pharmacokinetics analysis

There was a significant increase in drug serum concentration 1 h (0.3 μg/mL, *p* < .01, 95% CI 0.2–0.4) and 2 h (0.2 μg/mL, *p* = .002, 95% CI 0.061–0.27) following the first drug administration (Table 2). The determined AUC_(0–10h)_ was 1.1 ± 0.5 μg/mL*h, the MRT was 6.7 ± 4.3 h, and the *C*_max_ 0.4 ± 0.4 μg/mL. The *t*_max_ was 1.8 h (geometric mean). The serum concentration profile for the entire experimental period is shown in Table 2. The *C*_max_ peaked (highest value) at 72 h after the initiation of the therapy, indicating that the steady state was reached at 72 h after the first acetazolamide oral dose. There was a significant increase in drug serum concentrations 48 h (0.2 μg/mL, *p* = .005, 95% CI 0.05–0.27), 72 h (0.2 μg/mL, *p* = .02, 95% CI 0.02–0.2), and 156 h (0.1 μg/mL, *p* = .02, 95% CI 0.02–0.23) after initiation of treatment (Table 2). Serum drug concentration decreased on days 9 and 10 and is not statistically different from time zero (*p* > .05).

**TABLE 2:** The average acetazolamide serum concentrations in healthy horses before, during, and after acetazolamide treatment.

Time (h)	Acetazolamide serum concentrations μg/mL (95% CI)	*p*‐value
0^a^	0.08 (−0.07, 0.23)	
1	0.29 (0.19, 0.4)	**<.0001**
2	0.17 (0.06, 0.27)	**.002**
6	0.05 (−0.04, 0.16)	.34
10	0.02 (−0.08, 0.13)	.67
12	0.06 (−0.05, 0.16)	.3
24	0.09 (−0.01, 0.19)	.09
36	0.07 (−0.03, 0.17)	.21
48	0.15 (0.05, 0.26)	**.005**
72	0.17 (0.07, 0.28)	**.001**
156	0.13 (0.02, 0.23)	**.02**
204	0.05 (−0.05, 16)	.33
228	0 (−0.12, 0.1)	.962

*Note*: Bolded values were significant.

^a^ Time 0 was immediately before administration of the first acetazolamide dose and was considered the reference value.

Blood work at the end of the study period, including electrolytes, creatinine, and urea levels, was within normal limits for all horses.

## 4 DISCUSSION

In veterinary ophthalmology, systemic CAIs are infrequently used for glaucoma treatment in dogs and cats but, to our knowledge, have not been studied as potential glaucoma medication in horses. Acetazolamide is being used in equine medicine for HYPP treatment in adult horses and foals.^10^, ^11^ Our results indicate that systemic acetazolamide administration to healthy horses significantly decreased intraocular pressure in two treatment days. The decrease in IOP was less than 3 mmHg, which may not be clinically relevant for glaucoma treatment in horses. It must be emphasized that these were horses with normal IOP before the study, and the difference may be significantly larger in horses with glaucoma. Comparison between IOP on the last day of treatment and IOP after treatment discontinuation reveals a difference of more than 5 mmHg in both eyes (Table 1), indicating that treatment discontinuation caused a significant increase in IOP. Therefore, further clinical studies are required to determine whether acetazolamide is a potential treatment for equine glaucoma and, if so, to establish the correct doses and discontinuation protocol.

IOP decreased significantly on the third day of drug administration when drug serum levels reached a steady state. However, during the following 3 days (days 4–6), IOP increased back to baseline values measured during the adaptation period. This could be explained by the drug's poor and inconsistent absorption together with the effects of the “flip‐flop” pharmacokinetic pattern. This phenomenon occurs in extravascularly administered drugs when the rate of absorption is slower than the rate of elimination. “Flip‐flop” disposition can result from modified dosage formulations, chemical properties of the drug and the physiological makeup of extravascular drug administration.^12^ Therefore, these factors may affect the clinical outcome and explain the IOP fluctuations during the study.

The goal of this study was to investigate the effect of oral acetazolamide on IOP and to support the dose determintion. As indicated above, the intravenous and oral pharamacokinetics of acetazolamine in horses has been studied previously.^6^ However, the study by Alberts et al.^6^ described single‐dose pharmacokinetics. Therefore, and specifically because of the drug's “flip‐flop” kinetics, we aimed to determine the acetazolamide concentration during a treatment period of 1 week. The Alberts et al.^6^ study revealed that acetazolamide has a clear “flip‐flop” pharmacokinetic pattern after oral administration despite its short *t*_max_. Although not calculated in the original publication, the mean absorption time (MAT) appears to be approximately 5 h compared to the short MRT of 1.7 h after intravenous administration, indicating very slow absorption compared to elimination. Hence, the calculation of the drug's half‐life (*t*_1/2_) after oral administration is not helpful and is often misleading. The MRT_oral_ determined in our study after the first acetazolamide administration was almost identical to that of the Albert study (6.7 ± 4.3 and 6.8 ± 1.4 h, respectively). The steady state is reached after four half‐lives.^13^ Based on the Alberts et al.^6^ study, it would occur after 30 h. The bioavailability of acetazolamide in horses was found to be only 25% in the Alberts et al.^6^ study. The bioavailability was obviously even lower in the present study as the AUC and *C*_max_ reported here were less than half of those expected from the earlier study. The *C*_max_ is a more accurate marker as the AUC is determined differently (to infinity vs. to 10 h). Low bioavailability causes greater fluctuation in serum concentrations over time. It appears that the steady state was reached around 72 h after the first acetazolamide dose, which would be consistent with the acetazolamide *t*_1/2_ determined after intravenous administration and can be used as a guide in dose determinations. Cosidering the “flip‐flop” pharmacokinetics pattern, it would be appropriate to investigate the drug serum concentration and its' effect on IOP for different oral acetazolamide dosages and longer treatment periods.

Acetazolamide has some potential side effects. In humans, acetazolamide may cause paraesthesias, dysgeusia, polyuria, and fatigue.^14^ In newborn infants, metabolic acidosis was detected following maternal use of acetazolamide during pregnanacy.^15^ In excercising horses, chronic acetazolamide treatment had a significant effect on the fluid flux in the lungs,^16^ association with hypercapnoea, acidosis^7^ and decreased exercise duration time.^8^ Because the horse is an athletic animal, side effects and treatment duration should be carefully considered. In this study, we demonstrated that the treatment discontinuation caused an increase in IOP, which may warrant a gradual discontinuation of the drug. Also, the posttreatment increase in IOP was higher than the decrease in IOP during the treatment, a phenomenon that should be further invetigated as it may mean that the drug is contraindicated in glaucomatous horses.

The study had several limitations. First, we compared IOP during and after treatment to IOP measured prior to treatment. Therefore, there was a lack of true controls during the study period. In addition, the veterinarian testing the IOP was not blinded to the treatment. The bioavailability of oral acetazolamide is only 25%, which may influence the results. We chose to administer the drug orally to resemble a field treatment, but intravenous admisinistration might have led to more prominant results. In addition, the veterinarian testing the IOP was not blinded to the treatment, therefore, bias may have been inadvertently introduced into the results. Serum levels measured before medication was given for the first time were higher than zero for some horses due to measurement standard deviation, which may influence the results. The lack of information regarding the horses breeds (all were local breeds) may increase the variance in the studied population. In addition, we examined the effect of oral acetazolamide administration in healthy horses during 1 week of treatment. In order to demonstrate a more accurate clinical effect, the drug should be tested in glaucomatous horses. In addition, only one drug dosage, suitable for HYPP, was tested due to safety considerations. Considering the “flip‐flop” pharmacokinetics pattern, it would be appropriate to investigate the drug serum concentration and its effect on IOP for different acetazolamide dosages and longer treatment periods.

## 5 CONCLUSIONS

This present study tested IOP response to acetazolamide treatment during 1 week and revealed potential for lowering IOP, as well as a significant posttreatment IOP increase, in normal eyes. Further clinical study is required to determine whether acetazolamide is a potentially effective treatment for equine glaucoma.

## AUTHOR CONTRIBUTIONS

**Anat Shnaiderman‐Torban:** Conceptualization; data curation; investigation; project administration; visualization; writing – original draft. **Oren Pe'er:** Conceptualization; investigation; writing – review and editing. **Kajsa Gustafsson:** Investigation; writing – review and editing. **Amos Tatz:** Investigation; writing – review and editing. **Malka Brizi:** Formal analysis; resources. **Stefan Soback:** Conceptualization; formal analysis; methodology. **Wiessam Abu Ahmad:** Conceptualization; formal analysis. **Ramon Magen:** Investigation. **Ron Ofri:** Conceptualization; supervision; writing – review and editing. **Gal Kelmer:** Conceptualization; funding acquisition; supervision; writing – review and editing.

## CONFLICT OF INTEREST STATEMENT

None.

## Data Availability

Data available on request from the authors.
